# Selfing rates vary with floral display, pollinator visitation and plant density in natural populations of *Mimulus* *ringens*

**DOI:** 10.1111/jeb.13781

**Published:** 2021-03-27

**Authors:** Dorothy A. Christopher, Jeffrey D. Karron, Wendy R. Semski, Patrick A. Smallwood, Dorset W. Trapnell, Randall J. Mitchell

**Affiliations:** ^1^ Department of Biological Sciences University of Wisconsin – Milwaukee Milwaukee WI USA; ^2^ Department of Plant Biology University of Georgia Athens GA USA; ^3^ Department of Biology University of Akron Akron OH USA

**Keywords:** *Bombus*, floral display, geitonogamy, mating system, *Mimulus*, plant density, pollen limitation, pollination, pollinator visitation rate, selfing rate

## Abstract

Variation in selfing rates within and among populations of hermaphroditic flowering plants can strongly influence the evolution of reproductive strategies and the genetic structure of populations. This intraspecific variation in mating patterns may reflect both genetic and ecological factors, but the relative importance of these factors remains poorly understood. Here, we explore how selfing in 13 natural populations of the perennial wildflower *Mimulus ringens* is influenced by (a) pollinator visitation, an ecological factor, and (b) floral display, a trait with a genetic component that also responds to environmental variation. We also explore whether genetically based floral traits, including herkogamy, affect selfing. We found substantial variation among populations in selfing rate (0.13–0.55). Selfing increased strongly and significantly with floral display, among as well as within populations. Selfing also increased at sites with lower pollinator visitation and low plant density. However, selfing was not correlated with floral morphology. Overall, these results suggest that pollinator visitation and floral display, two factors that interact to affect geitonogamous pollinator movements, can influence the selfing rate. This study identifies mechanisms that may play a role in maintaining selfing rate variation among populations.

## Graphical abstract

Selfing rates vary widely among natural populations of the perennial wildflower Mimulus ringens. Increased selfing is positively correlated with floral display size and negatively correlated with pollinator visitation and plant density. This suggests that among‐population variation in selfing rate reflects the interaction between ecological and genetic factors.

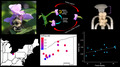

## 1 INTRODUCTION

Most flowering plants are hermaphroditic and have the potential to reproduce sexually through both self‐ and cross‐fertilization. The extent of cross‐fertilization varies widely within and among populations and may have important consequences for spatial genetic structure, patterns of gene flow and the magnitude of inbreeding depression (Barrett & Harder, 2017; Devaux et al., 2014; Whitehead et al., 2018). Although considerable theoretical work has explored the conditions that favour the evolution of self‐ and cross‐fertilization, the mechanisms leading to these evolutionary trajectories are less well understood.

Among‐population variation in selfing rate may result from both genetic and ecological mechanisms (Barrett & Harder, 1996; Devaux et al., 2014; Koski et al., 2019). Genetic factors affecting selfing include the transmission advantage of selfing, inbreeding depression and differences among populations in heritable floral traits that favour selfing (such as the proximity of anthers to stigma). Ecological factors affecting selfing include population density and size, plant floral display size, pollinator abundance and behaviour, pollinator sharing with co‐flowering species and pollen limitation (Barrett & Eckert, 1990; Devaux et al., 2014; Schemske & Lande, 1985). Additionally, population history and geographic location may also be associated with differences in selfing rate because individuals that can self will not experience mate limitation and so have an increased ability to found new populations and expand the species range (Grossenbacher et al., 2015; Koski et al., 2019; Moeller et al., 2017). Ultimately, the combined and potentially interacting effects of these factors on pollination dynamics determines the selfing rate (Cruzan & Barrett, 2016; Johnston et al., 2009; Sorin et al., 2016). Pollination dynamics include receipt of outcross pollen, receipt of self‐pollen, export of pollen to conspecifics and any potential post‐pollination effects such as pollen competition, stylar screening and zygote vigour.

In natural populations, the genetic and ecological factors influencing selfing rate frequently co‐vary, and therefore, it is often difficult to ascertain the mechanisms responsible for among‐population variation in selfing (Koski et al., 2019; Whitehead et al., 2018). Experimental studies suggest that floral display could play an important role, since pollinators tend to visit more flowers sequentially on large displays (Mitchell et al., 2004; Robertson, 1992), increasing the extent of among‐flower, within‐plant (geitonogamous) self‐fertilization (Devaux et al., 2014; Karron et al.,. 2004, 2009). However, the influence of floral display on among‐population variation in selfing rates has seldom been quantified in natural populations (Brunet & Sweet, 2006; Koski et al., 2019). This may, in part, relate to the challenge of measuring floral display—a dynamic trait that varies from day to day, and therefore requires regular evaluation of known plants across the flowering season, or calibration of covariates that are associated with daily display (Williams, 2007).

Here, we explore how genetic and ecological factors influence selfing rate in natural populations of the perennial wetland plant *Mimulus ringens* L. (Phrymaceae). In particular, we address three questions:

1. Does *M. ringens* selfing rate vary among populations?
2. Does population mean selfing rate vary with floral display?
3. What floral traits and habitat factors are associated with selfing rate?

## 2 METHODS

### 2.1 Study system

*Mimulus ringens* (Phrymaceae) is an herbaceous wetland perennial native to central and eastern North America. Individual plants produce one, or occasionally several stems that flower in July and August and exhibit very local clonal reproduction across years. The large (20–30 mm length) zygomorphic flowers are blue‐purple in colour and last for half a day. Plants are fully self‐compatible. Pollination is primarily by large bees, especially *Bombus* spp., that seek both nectar and pollen (Mitchell et al., 2004). These floral rewards are available to visitors upon anthesis (which occurs before dawn) and are not renewed following visitation, so standing crops decline monotonically over the morning hours (unpublished data). Stigma closure following pollination of *M. ringens* does not exhibit the rapid (3–12 s) closure shown by many other members of the genus (Beardsley & Barker, 2005; Fetscher & Kohn, 1999), but stigmas do close slowly (3–300 min) and permanently in response to pollen delivery following a single visit (Mitchell et al., 2005; unpublished data). All flowers produce a fruit, which can contain up to 4,000 seeds. To our knowledge, there is no early acting inbreeding depression in this species (Sorin et al., 2016, unpublished data).

We studied 13 natural populations in northeastern Ohio, USA. Populations ranged from 5 to 85 km apart. We chose populations based on their expected plant mean daily floral display, with the goal of including a large range of mean display sizes among populations. We informed our choices with data from prior work, and visual assessment of plant size before flowering (early July 2018). We only considered populations occupying at least 100 m^2^ (or containing > 200 genets) in order to ensure enough space and plants for the field work. Most sites were in wet meadow habitats, and several were adjacent to active beaver ponds. Dominant vegetation primarily consisted of species with facultative or obligate wetland indicator status (77/90 species, based on classifications in Andreas et al., 2004), such as *Typha*, *Sparganium* and *Phalaris*. The most common co‐flowering species that shared pollinators with *M. ringens* at these sites were *Verbena hastata*, *Eutrichium perfoliatum*, *Impatiens capensis* and *Asclepias incarnata*.

### 2.2 Characterization of floral display

At each site, we identified 71–81 *M. ringens* focal plants for characterizing flowering patterns and for collecting fruits for mating system study. To do this, in each population we established 3–10 parallel transects, each 1 m wide, with 0.5 m separation between transects. Transects were typically 20–30 m long, and the maximum distance between focal plants at a site averaged 36 m. During June and July 2018, we evaluated each 1 m square along each transect, counting all *Mimulus* stems and genets. To distinguish individual genets, we used spacing between stems, evidence of underground connections and vegetative morphology. In each square, we chose and labelled as a focal plant the *M. ringens* stem closest to the centre (therefore using only one stem per genet). In one population (LNB), we could only find 71 suitable focal genets. The transects provide estimates of stem and genet density for each population and included a large fraction of all plants in most populations. To assess total population size, we combined those counts with a visual estimate of the number of plants not on the transects.

We evaluated daily floral display (number of open flowers) for each focal stem for each population about twice a week throughout the flowering season (5–9 times over 39 days). Flowering at a site lasted a mean of 30.5 days (range = 23–36 days). First flowering for these populations occurred between 15 July and 27 July.

To characterize daily floral display for each population across the season, we used the maximum daily floral display for each plant across floral census dates and calculated the mean across the 71–81 focal plants at each site (these values are approximately normally distributed). We refer to the population mean of these values as the ‘mean‐maximum daily floral display’. After flowering concluded, we returned to each population and counted all fruits produced on focal plants. Since all flowers produce fruits in *M. ringens*, this also documented ‘total flower production’, which we used as a second index of floral display.

Near the flowering peak for each population, we measured floral morphology for 28 separate genets (carefully avoiding the focal plants, so that their pollination was not altered). Each flower came from a separate *M. ringens* stem, separated from any other sampled stem by >1 m. Because floral traits might change over the day and in response to visitation, we began measurements early in the morning (7–8 a.m.), before pollinators became active. We used digital calipers to measure: corolla width (greatest horizontal distance across petals), corolla height (greatest vertical distance across petals), tube length (distance from base of calyx to the sinus between the upper and lower petal lobes), style length (distance from base of calyx to tip of the open stigma) and herkogamy (shortest distance from anthers to stigma surface; Bodbyl Roels & Kelly, 2011).

### 2.3 Pollen limitation

We tested for pollen limitation by comparing seed production of control flowers to that of flowers receiving supplemental outcross pollen. To do this, immediately after measuring floral morphology we haphazardly chose 25 flowering stems along the transect lines. All chosen stems were separated by >1 m from one another and were not focal plants. If the stem had two or more flowers, we randomly chose one flower to receive supplemental pollen. We applied pollen from other plants >1 m away by stroking a fresh anther over the stigma surface. We then repeated this using an anther from a different plant and labelled both the pollinated and control flowers for later harvest. The ‘control’ flower was typically at the same node as the ‘supplemented’ flower and was handled in the same way but did not receive supplemental pollen. We repeated this for 25 stems in each population. Because closed stigmas cannot receive additional pollen, we did not pollinate or use as controls the few randomly chosen flowers that had closed stigmas. Four to five weeks after pollination we collected ripe fruits from these flowers. In some populations, a substantial fraction of fruits was damaged by small caterpillars (Verbena Bud Moth, *Endothenia hebesana*; Tortricidae), preventing seed counts. For undamaged fruits, we counted the number of seeds produced. To facilitate accurate and speedy counting of the minute and numerous seeds of *M. ringens,* we used a flatbed scanner and computer. We placed the seeds from each fruit into a separate clear locking sandwich bag to facilitate handling and scanned at 600 DPI, using image‐J software (Schneider et al., 2012) to count the number of seeds / fruit. We repeated this three times for each bag (repositioning seeds between scans) and used the mean of these counts in analysis (scan counts match hand counts closely: *r* = 0.97, *N* = 20). We used population means for each pollen limitation treatment to calculate a pollen limitation index at each population: PL = (supplemented – control)/control (Eckert et al., 2010; Koski et al., 2017).

To quantify the proportion of flowers with open stigmas across the 6‐hr pollination window, we periodically walked the transect lines and inspected at least 30 haphazardly selected flowers separated by at least 1 m from one another. We scored as ‘open’ any stigma that showed no indication of stigma closure (reduction in the angle between the upper and lower lobes), even if there was pollen visible on the open stigmatic lobes. We used stigma scores from the floral morphology and pollen limitation surveys to supplement these data. We continued observations until most stigmas were closed, which typically occurred by noon.

### 2.4 Pollinator visitation

We quantified pollinator visits to *M. ringens* flowers on 1–2 days in each population, immediately after floral measurements and at intervals throughout the morning. To do this, we observed patches of 20–100 flowers and recorded all floral visits by each visitor taxon during a 15‐min observation period. We considered legitimate visitors to be those that entered flowers and contacted anthers and stigma. We identified large pollinators to species (e.g. species of *Bombus*) but could confidently identify smaller visitors (e.g. *Ceratina*, *Augochlora*, *Lasioglossum*) only to genus. We considered as pollinators the larger bees (~10 mm length and larger; in this study, they include several species of *Bombus*, *Apis mellifera*, *Xylocopa virginica* and *Anthophora terminalis*) based on our frequent observations of them visibly transferring pollen. We considered all other visitors to be nonpollinators; this includes those that did not contact reproductive parts (mostly nectar robbing *Xylocopa*), as well as ineffective visitors such as Lepidopterans and small bees. This categorization is based on the small amounts of pollen carried by Lepidopterans, and the small amount of pollen transferred to *M. ringens* stigmas by small bees (unpublished data). We quantified visitation for at least one day in each population and obtained a second day of observation in 10 populations. We observed visitation approximately once an hour beginning at 8–10 a.m. (after completing the time‐sensitive morphology and pollen limitation work), continuing until closure of most stigmas prevented effective pollination (typically the majority of stigmas were closed before noon). Most days there were two observers, each observing a separate patch of flowers during each period. We accumulated between 4 and 15 separate observation periods in each population, for a total of 25 hr of observation. We performed an analysis of variance to determine whether the visitation rates between pollinating and nonpollinating insects differed significantly. We calculated the correlation between pollinators and nonpollinators within an observation period.

### 2.5 Seed collection and seedling genotyping

To assess the selfing rate for each population, we collected 10 fruits from each of 20 randomly selected maternal focal plants in each population. We excluded any focal plants that had fewer than 10 fruits. After drying fruits for two weeks, we extracted and bulked seeds from all fruits on a focal stem.

To genotype seedlings, we germinated 10 seeds from the bulked collection from each maternal plant in separate pots. Germination rates were >80% for all populations. At two weeks post‐germination, seedlings were transplanted in 10 cm pots to grow for an additional two weeks. We harvested one leaf per seedling for genotyping. We extracted DNA following a modified CTAB protocol (Doyle, 1991). We genotyped the seedlings at eight microsatellite loci following Nunziata et al., (2012). We genotyped 20 maternal families per population with 10 seedlings per maternal family. The average amount of missing data was 3% across populations.

### 2.6 Mating system analyses

To estimate the multilocus selfing rate of *M. ringens* in each population, we used MLTR v3.2 (Ritland, 2002). We retained the default MLTR parameters, which constrained gene frequencies to equal ovule frequencies, and calculated standard errors using 10,000 bootstrap replicates with the maternal family as the resampling unit. We also estimated the selfing rate separately for plants with large floral displays and small floral displays within a population. We divided the 20 maternal plants within a population into two groups, the 10 plants with the largest displays and the 10 plants with the smallest displays. We then ran MLTR separately on these two groups using the same parameters as above.

Previous work has demonstrated that *M. ringens* exhibits biparental inbreeding and correlated matings (flowers that receive one pollinator visit have approximately three sires per fruit; Christopher et al., 2019; Karron et al., 2006). Although these factors may bias the estimate of the outcrossing rate, MLTR accounts for them by using a correlated‐matings model that takes a progeny pair as the unit of observation (Ritland, 2002). We sampled 10 offspring per maternal family, thus avoiding problems with estimation bias that occur for smaller samples (Koelling et al., 2012).

We estimated the adult inbreeding coefficient *F* using BORICE v1.1 (Koelling et al., 2012). We then calculated inbreeding depression using the formula from Ritland (1990):

$$\delta =1‐2\left[\frac{(1‐s)F}{s(1‐F)}\right].$$

### 2.7 Statistical analyses

We used population means for all traits and population‐level selfing rate estimates in the analyses (*N* = 13 populations). The exception is flower production, for which we used total flower production of each of the 20 maternal genets sampled for selfing rate estimation in each population as an index of daily floral display. We log transformed the floral display data; this transformation was selected based on AIC scores between alternative transformations.

We used model selection to evaluate which measured variable(s) best predict the selfing rate (Burnham & Anderson, 2002). To assess and quantify the relationship between the variables and selfing rate, we fit generalized linear models. We had strong a priori hypotheses about the importance of floral display, and therefore all models include a floral display term. Additional predictor variables include: pollinator visitation rate, plant density, herkogamy, population size (plant number), nonpollinator visitation rate and flower size. Because floral display, density and pollinator visitation rate were significant, we tested one model that included all three of these variables. We used an information theoretic approach (AIC) and the Akaike second‐order information criterion (AICc) to select the best model (model with the lowest AICc score). We calculated ΔAICc by subtracting the AICc score of each model from the model with the lowest AICc. Models with ΔAICc less than 2 are substantially supported (Burnham & Anderson, 2002). AICc was calculated using R package MuMIn (Barton, 2015).

We also investigated the relationship between floral display size and selfing within a population. We used a paired *t*‐test to determine whether the group of 10 plants with large displays had higher selfing rates than the 10 plants with small floral displays. Statistical analyses were performed using R v3.6.1 (R Core Team, 2019) and SAS/STAT^®^ 9.4 software (SAS Institute, 2013).

## 3 RESULTS

The 13 field populations of *M. ringens* across northeastern Ohio varied widely in size and density (names and locations in Table S1). Population size varied from 250 to 10,000 genets, and density ranged from 0.54 to 10.9 genets/m^2^ (Table 1).

**TABLE 1:** Population characteristics

Pop. code	Pop. size	Genets/m^2^	Mean‐maximum floral display (mean/stem)	Mean total flower production/stem	N Pollinator Observation Periods
CBC	450	1.09 ± 0.15	2.4 ± 0.4	29 ± 4	8
ECM	3,000	10.88 ± 1.05	1.5 ± 0.2	19 ± 2	12
HBP	900	3.12 ± 0.37	2.2 ± 0.4	21 ± 3	9
LEB	500	0.98 ± 0.12	4.2 ± 0.7	45 ± 10	8
LIB	600	1.44 ± 0.21	4.7 ± 0.9	69 ± 14	7
LNB	250	0.54 ± 0.07	3.6 ± 0.8	56 ± 20	4
MCW	1,000	0.57 ± 0.09	6.0 ± 1.2	61 ± 18	15
MSB	900	0.99 ± 0.12	7.5 ± 1.3	85 ± 14	6
RIS	900	1.84 ± 0.21	4.7 ± 1.0	67 ± 15	10
SKO	10,000	8.87 ± 0.91	3.8 ± 0.7	46 ± 8	8
STR	450	1.65 ± 0.21	2.7 ± 0.6	27 ± 4	5
WBW	950	2.31 ± 0.24	4.1 ± 0.8	54 ± 9	4
WET	300	0.72 ± 0.09	2.9 ± 0.4	32 ± 5	4

Note

Population sizes were visually counted. Density estimates include mean and *SE* based on 89–309 1 m^2^ plots. Mean‐Maximum Floral Display is the mean of the maximum observed daily floral displays across 5–9 census dates during the flowering season. Total Flower Production is the mean per stem from direct counts at season's end. Both display measures are based on 81 genets/population (except for site LNB, where *N* = 71). *N* Pollinator Observation Periods refers to the number of observation periods conducted in each population.

Floral displays varied greatly among populations (Table 1) in both the mean of maximal daily displays per stem (range = 1.5 to 7.5 flowers/stem), and in total fruit production (range 21–85). A nested variance component analysis on mean‐maximum floral display indicates 85.3% of the variation was within populations, and 14.7% was among populations. Both measures of display were strongly correlated with one another (*r* = 0.92, *p* <.0001). Both measures are based on 71–81 focal stems/population; however, note that in the selfing rate analyses below, we used floral display data from only the 20 plants genotyped to estimate the mating system.

Floral morphology varied significantly and substantially among populations for all measures (Table 2). These traits showed notable covariation (Table S2). A principal components analysis revealed two significant axes of variation. Axis one loaded strongly on petal and style characters, whereas axis two primarily reflected variation in herkogamy.

**TABLE 2:** Population mean floral morphology and principal component loadings

Population	Corolla width	Corolla height	Tube length	Style length	Herkogamy
CBC	21.87 ± 0.41	18.80 ± 0.27	17.80 ± 0.16	19.86 ± 0.20	1.57 ± 0.12
ECM	22.85 ± 0.29	18.62 ± 0.24	19.25 ± 0.18	20.67 ± 0.23	1.30 ± 0.11
HBP	21.99 ± 0.46	17.78 ± 0.27	18.62 ± 0.18	20.16 ± 0.14	1.58 ± 0.12
LEB	24.60 ± 0.32	19.31 ± 0.26	20.41 ± 0.28	22.21 ± 0.20	1.99 ± 0.09
LIB	17.82 ± 0.53	16.24 ± 0.37	17.61 ± 0.19	20.19 ± 0.22	1.94 ± 0.11
LNB	20.06 ± 0.28	14.13 ± 0.30	17.54 ± 0.16	20.61 ± 0.18	2.13 ± 0.12
MCW	21.67 ± 0.38	17.66 ± 0.26	17.95 ± 0.18	20.64 ± 0.19	1.74 ± 0.09
MSB	21.43 ± 0.41	18.87 ± 0.29	19.52 ± 0.22	21.73 ± 0.19	1.72 ± 0.11
RIS	22.44 ± 0.47	19.15 ± 0.19	18.75 ± 0.16	20.07 ± 0.15	0.63 ± 0.09
SKO	21.79 ± 0.27	17.90 ± 0.25	18.38 ± 0.20	19.92 ± 0.20	0.94 ± 0.05
STR	23.17 ± 0.30	18.09 ± 0.19	19.40 ± 0.21	21.23 ± 0.19	1.12 ± 0.06
WBW	22.97 ± 0.31	17.25 ± 0.32	19.52 ± 0.26	20.80 ± 0.28	1.01 ± 0.08
WET	21.93 ± 0.29	16.72 ± 0.24	18.03 ± 0.16	20.24 ± 0.22	2.13 ± 0.10
*F*	**18.9**	**27.5**	**23.6**	**13.2**	**21.9**
Variation among populations (%)	39	49	45	30	43
Axis 1	0.751	0.743	0.888	0.723	−0.181
Axis 2	−0.207	−0.165	0.077	0.572	0.902

Note

*N* = 28 genets/Population, 1 flower/genet. *F* tests from ANOVA are for population differences (*df* = 12, 350). Significant *F* values (*p* <.0001) are presented in bold. Values are LSmeans ± *SE* (mm). Loadings are from a principal component analysis with varimax rotation. Axis 1 accounts for 49.2% of variation, Axis 2 accounts for an additional 24.3%.

During 100 observation periods in our 13 populations, we documented 721 individual visitors, mostly bees (98.6%). Over 75% of legitimate pollinators to *M. ringens* were *Bombus*, and over half of the *Bombus* were *B. impatiens* (57.8%). The remainder were *B. fervidus* (12.1%), *B. vagans* (4.2%), *B. griseocollis* (1.8%) and *Bombus* that could not be identified to species (<1%). Other large bee pollinators included *A. mellifera* (20.0%; only at two sites), *A. terminalis* (2.4%) and *X. virginica* visiting legitimately (1.2%). Over 3/4 of individual visitors to *M. ringens* were nonpollinators. The vast majority (67%) were small bees, including *Lasioglossum*, *Augochlora* and *Ceratina*. Robbing *Xylocopa* accounted for 6%, and another 1.4% were Lepidoptera, including the hawkmoths *Hemaris thysbe* and *Hemaris diffinis*, and some skippers (Hesperiidae). Individual pollinating bees (*Bombus* and large bees) typically visited many more flowers during a foraging bout than did other visitors, so pollinating bees accounted for nearly half (48.9%) of the 2,369 recorded flower visits.

**FIGURE 1:**
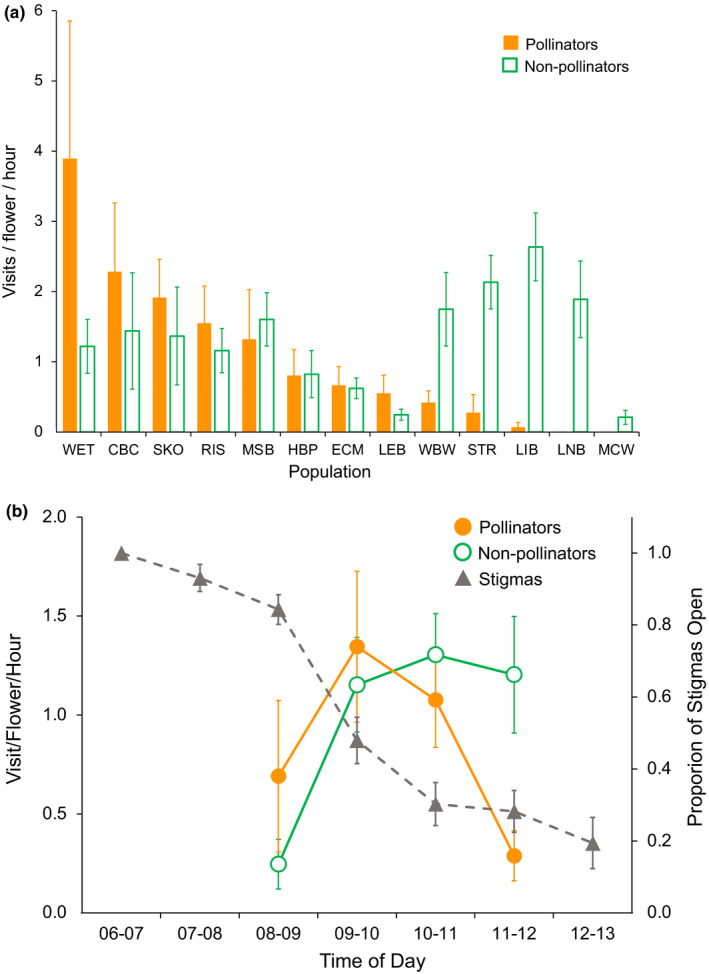
(a) Flower visitation rate by pollinators and nonpollinators for each population. Populations are ranked by visitation rate for pollinating bees. (b) Flower visitation to *Mimulus ringens* during timed observations and patterns of stigma closure. Pollinators include large bees (*Bombus*, *Apis*, *Anthophora*) and nonpollinators include small bees, nectar robbers and Lepidopterans. Visitation based on 100 observation periods of 15 min each across the 13 populations. Values shown are LS Means with *SE*. Proportion of stigmas open is based on surveys of >24 flowers per time period at each of the 13 populations

Flower visitation during timed observation periods varied significantly among populations, both for pollinators (large bees like *Bombus*, *Apis* and *Anthophora*; *F*_12,84_ = 3.79, *p* <.0001; Figure 1) and nonpollinating insects (small bees, nectar robbing bees and butterflies; *F*_12,84_ = 3.04, *p* <.001; Figure 1). There was no relationship between pollinator and nonpollinator visitation rates during an observation period (*r* = −0.08, *p* >.7). However, the time of visitation differed strongly between those two groups (Figure 1b). Pollinators showed a marked peak in visitation during the mid‐morning hours, coinciding with stigma closure. By contrast nonpollinators showed a plateau that held steady through the later observations (noon), well after the majority of stigmas had closed. The abundance of pollinating bees varied widely among populations, but most populations had substantial visitation by nonpollinators (Figure 1a). Open stigmas declined rapidly over the morning at all sites, although the rate varied greatly. The mean time for at least 50% closure was 10:14 a.m. (*N* = 23), ranging from 7:50 to noon, and closely matched the time of increased visitation by pollinators.

Pollen limitation significantly decreased with increased pollinator visitation (*F*_1,11_ = 6.17, *R* ^2^ = 0.301, *p* =.03). However, the overall amount of pollen limitation across populations was low (mean ± *SE* =0.047 ± 0.269; range from −0.12 to + 0.27). Pollen limitation was not influenced by nonpollinator visitation (*F*_1,11_ = 0.4, *p* >.5). Seed production for open pollinated flowers from the pollen limitation study varied significantly among populations (*F*_12,367_ = 12.54, *p* <.0001), with means ranging from 1,925 to 3,714 seeds/fruit. Seed predation (% fruits damaged) varied greatly among populations, from 0% to 88% (*N* = 47–50 genets / population). Rates of damage were not significantly correlated with pollinator visitation (*r* = 0.03, *p* =.67), nonpollinator visitation (*r* = −0.06, *p* =.53) or pollen limitation (*r* = −0.32, *p* =.09).

### 3.1 Variation in selfing rates among populations

**FIGURE 2:**
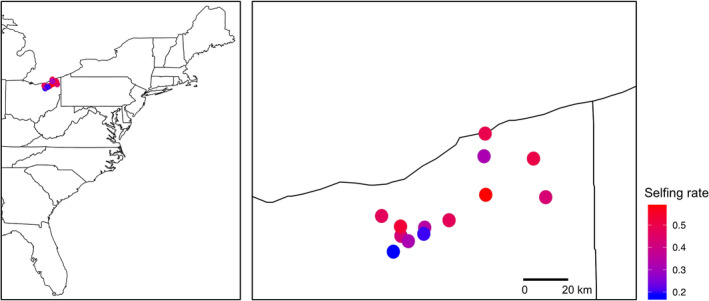
Geographical locations of 13 populations and associated selfing rates for *Mimulus ringens* populations in northeastern Ohio, USA. Left panel shows locations of the populations on a map of the eastern United States, and the right panel shows a detailed map of the populations in northern Ohio

The 13 populations varied widely in selfing rate (*s*), ranging from 0.13 to 0.55 (Table 3), with an overall mean of 0.38 ± 0.03. There was no spatial pattern to this selfing rate variation (Figure 2). The mean inbreeding coefficient for adult plants (F) across populations was 0.13 ± 0.02, ranging from 0.05 to 0.26.

**TABLE 3:** Mating system summary statistics for 13 natural populations of *Mimulus ringens*, ±*SE*

Population	*s*	*F*	95% CI for *F*	*δ*
CBC	0.30 ± 0.01	0.10	0.03–0.18	0.48
ECM	0.13 ± 0.01	0.06	0.00–0.14	0.15
HBP	0.38 ± 0.02	0.14	0.03–0.25	0.47
LEB	0.51 ± 0.02	0.15	0.07–0.25	0.66
LIB	0.46 ± 0.01	0.13	0.04–0.24	0.65
LNB	0.55 ± 0.02	0.26	0.14–0.40	0.42
MCW	0.44 ± 0.02	0.05	0.00–0.12	0.85
MSB	0.49 ± 0.01	0.09	0.03–0.17	0.79
RIS	0.52 ± 0.01	0.18	0.07–0.28	0.59
SKO	0.28 ± 0.02	0.17	0.09–0.26	−0.05
STR	0.48 ± 0.03	0.12	0.05–0.20	0.70
WBW	0.31 ± 0.01	0.20	0.10–0.31	−0.11
WET	0.17 ± 0.01	0.07	0.00–0.14	0.26

Abbreviations: F, inbreeding coefficient and 95% credible interval; s, selfing rate; δ, inbreeding depression.

**FIGURE 3:**
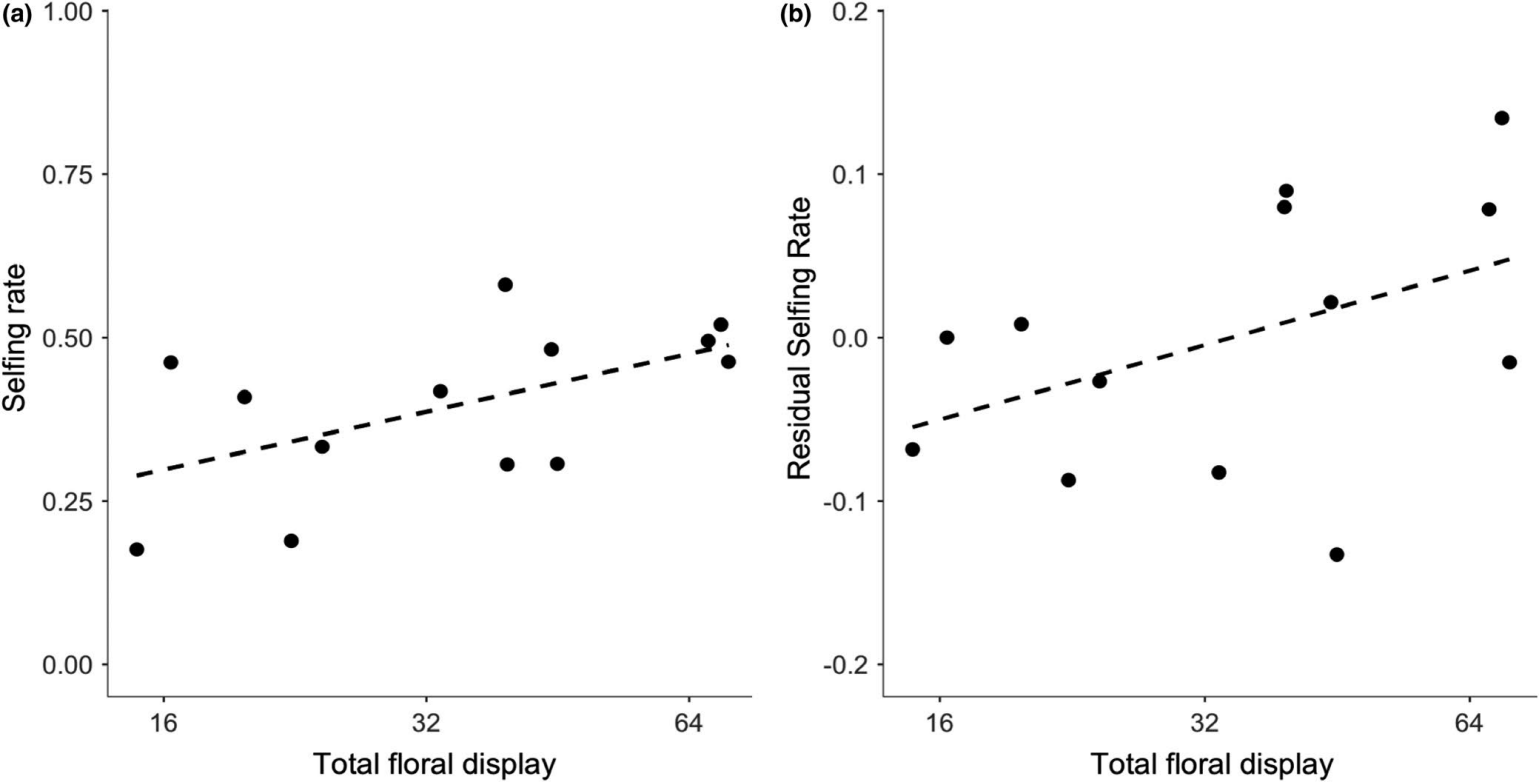
Selfing rate, floral display and pollinator visitation rate. (a) Association between the selfing rate and total floral display for the 20 focal plants in 13 populations of *Mimulus ringens*. (b) Association between total floral display and the residual selfing rate (*y*‐axis) from a regression of pollinator visitation rate and plant density. Values are population means ± *SE*. Fitted line shows the regression slope. Note log scaling for *x*‐axis in a and b

Floral display size, pollinator visitation rate and plant density affected population selfing rate. Four models had ΔAICc < 2, and they shared the same predictors (Table 4). The most complex supported model included floral display, plant density and pollinator visitation rate (*R* ^2^ = 0.58, *p* =.01). The second best model included floral display and pollinator visitation rate (Figure 3 and Table 4, *R* ^2^ = 0.42, *p* =.028). Models with other predictors, including herkogamy, population size, pollen limitation, nonpollinator visits and flower size, were not supported (Table 4).

**TABLE 4:** Model selection

Model	*R* ^2^	*p*	AIC	AICc	ΔAICc	Weight	ER
Display + Visits +Density	**0.58**	**0.01**	**−62.1**	**−14.63**	**0**	**0.231**	**1**
Display + Visits	**0.42**	**0.028**	**−58.1**	**−14.52**	**0.11**	**0.219**	**1.06**
Display	**0.32**	**0.049**	**−55.87**	**−14.31**	**0.32**	**0.197**	**1.17**
Display + Density	**0.36**	**0.04**	**−57.37**	**−13.48**	**1.15**	**0.130**	**1.78**
Display + PL	0.31	0.06	−56.39	−12.50	2.13	0.080	2.90
Display + Population size	0.30	0.07	−56.15	−12.26	2.37	0.070	3.27
Display + Herkogamy	0.19	0.14	−54.17	−10.28	4.35	0.030	8.80
Display + NP Visits	0.18	0.15	−54.04	−10.14	4.49	0.024	9.44
Display + FS	0.17	0.15	−53.89	−10.00	4.63	0.022	10.12

Note

All models evaluated in the model selection, predicting the relationship between the measured variables and population selfing rate. ΔAICc is the difference in AICc between each model and the model with the lowest AICc. Best supported models (including models in which ΔAICc is <2.0) in bold.

Abbreviations: Display, total floral display; Visits, pollinator visitation rate; Density, number of genets per m^2^; PL, pollen limitation; NP Visits, nonpollinator visitation rate; FS, floral axis 1, population mean loading of the first principal component calculated using traits in Table 2.

**FIGURE 4:**
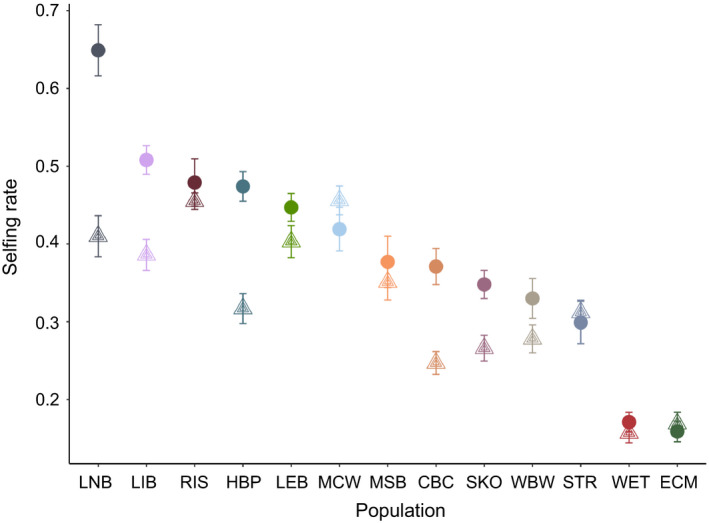
Within‐population differences in selfing rate for floral display groups. Values are mean ± *SE* selfing rate estimates for the 10 plants with larger floral displays, and the 10 plants with smaller displays in each population. Points are coloured by population; circles represent plants with large displays and triangles represent plants with small displays. *X*‐axis is sorted by selfing rate

To investigate the effect of floral display in more detail, we estimated selfing rate separately for two groups of plants in each population—the 10 plants with the largest displays (mean total display size 73 ± 9), and the 10 plants with the smallest displays (mean total display size 20 ± 2) (Figure 4; Table S3). The overall mean selfing rate for plants with larger displays is 0.39 ± 0.04, compared to 0.32 ± 0.03 for those with smaller displays. In 10 of 13 populations, the selfing rate was larger for the plants with larger displays, a significant difference using a paired *t*‐test (*t* = 2.90, *p* =.01).

**FIGURE 5:**
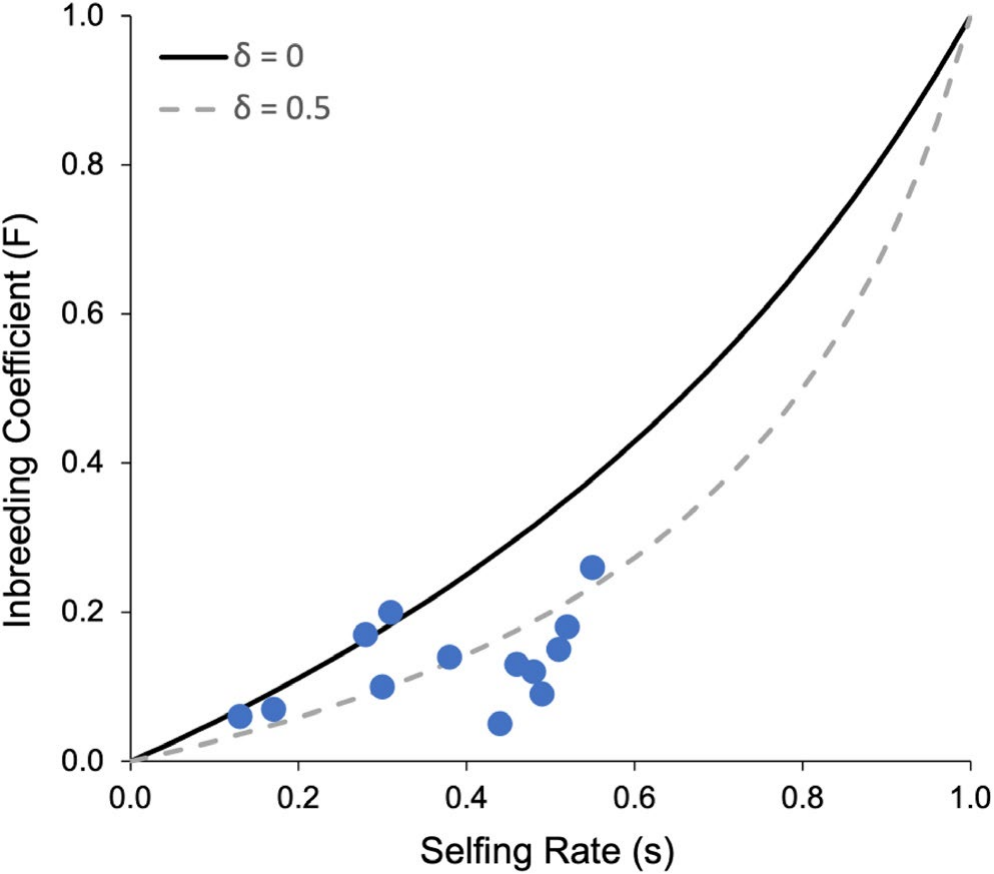
Relation between selfing rate and inbreeding coefficient. The orange solid line indicates the expected relationship in populations at equilibrium, with no inbreeding depression. The grey dashed line shows the equilibrium F if selfed progeny have 50% inbreeding depression (near the observed mean for these populations). Based on Goodwillie et al., 2005

Populations with higher selfing rates tended to have higher inbreeding coefficients for adult plants (*F* values), although this was not statistically significant (*R* ^2^ = 0.18, *p* =.08; Figure 5), but *F* values were usually much less than would be expected based on the selfing rate if there were no inbreeding depression. Inbreeding depression (δ) estimated with the Ritland (1990) method was substantial, with a mean of 0.45 ± 0.07, and values ranging from −0.11 to 0.85 (Table 3).

## 4 DISCUSSION

Plant mating systems often exhibit wide variation among species and among populations. Understanding the causes of this mating system variation requires examining many potentially important ecological and genetic factors. In this study, selfing rates in natural populations of *M. ringens* showed substantial variation across populations and were significantly affected by floral display, pollinator visitation and plant density. Interestingly, the selfing rate was not correlated with herkogamy or other heritable floral characters often thought to be associated with selfing. Finally, genetically inferred inbreeding depression was highly variable among the 13 populations. We discuss each of these results in detail below.

### 4.1 Variation in selfing rate and inbreeding depression among populations

Studies that explore patterns of selfing rate variation among populations are critical for understanding how the mating system influences evolutionary change. Historically, the selfing rate for an entire species is generally characterized using data from only a small number of populations. However, we found selfing rates among nearby populations of *M. ringens* varied widely, from 0.13 to 0.55. With so much variation, it is difficult to generalize about the selfing rate for an entire species (Whitehead et al., 2018). This study highlights that many factors can influence the selfing rate; therefore, understanding evolutionary responses to selfing may require a population‐by‐population evaluation of differences in ecological context, genetic structure, patterns of gene flow, strength of selection and their interactions (Barrett & Harder, 1996, 2017; Koski et al., 2019; Whitehead et al., 2018).

We found substantial variation among populations in our estimates of inbreeding depression, from −0.15 to 0.85, which spans a large portion of the range of possible values. It is important to note that this method of estimating inbreeding depression from parental and offspring *F* values assumes that selfing is the only cause of inbreeding depression (Ritland 1990; Goodwillie et al., 2005). Theory predicts that in populations with high selfing rates, inbreeding depression should be low because selection can, over time, eliminate deleterious alleles when homozygous (Byers & Waller, 1999; Charlesworth et al. 1990). However, we found that populations with high selfing rates had high inbreeding depression (Table 3), suggesting that these populations have not undergone purging of genetic load. In fact, our study joins a body of literature that has identified populations of many species with moderate to high selfing rates that nonetheless exhibit high inbreeding depression (e.g. Delmas et al., 2014; Eckert & Barrett, 1994; Herlihy & Eckert, 2002, 2005; Michalski & Durka, 2007; Tamaki et al., 2009). This may be due to the genetic architecture of inbreeding depression: weakly deleterious alleles are difficult to purge (Tamaki et al., 2009). Additionally, inbreeding may be a consequence of geitonogamous selfing resulting from large displays (Herlihy & Eckert, 2005). Understanding the relationship between selfing rate and inbreeding depression is important because high inbreeding depression should select for reduced selfing, and therefore inbreeding depression values can help explain the variation among population selfing rates.

### 4.2 Effects of floral display

Selfing rate increased significantly with floral display size among as well as within populations of *M. ringens* (Figure 5). Among populations, a threefold increase in floral display increased the selfing rate by approximately 30%, and within populations the plants with larger displays had higher selfing rates than plants with smaller displays. This response is probably caused by an increase in geitonogamous selfing when plants present more flowers at once. Larger floral displays are expected to increase selfing because they encourage among‐flower within‐plant (geitonogamous) pollinator foraging movements (Devaux et al., 2014; Harder & Barrett, 1995; Mitchell et al., 2004; Robertson & Macnair, 1995). Prior work on experimental arrays of *M. ringens* (Karron & Mitchell, 2012) linked geitonogamous pollinator movements directly to the selfing rate, and our current findings suggest that this relationship occurs in natural populations as well.

Very few studies have explored the relationship between selfing rate and floral display among natural populations (Brunet & Sweet, 2006; LoPresti et al., 2018). Our results are similar to those studies, which found a positive correlation between selfing and floral display, which they interpreted as an unavoidable consequence of larger displays to attract bumblebee pollinators for cross‐pollination. One reason for this similarity may be that bumblebees were pollinators in all of these studies; bumblebees often make short intraplant movements that encourage selfing. It would be informative to investigate selfing rates in a plant whose pollinators do not exhibit this geitonogamous visitation behaviour, or exhibit it to a lesser extent. Although it can be difficult to estimate the influence of floral display on selfing rates in natural population (Williams, 2007), floral display and the resulting geitonogamous selfing may be important in many species and therefore deserve more empirical consideration.

Among‐population variation in floral display may arise from both ecological and genetic causes. In experimental settings *M. ringens* floral display responds readily to resource availability (personal observation). However, differences among populations in total flower production and in how flowers are deployed over the flowering season may also have a genetic component (Karron & Mitchell, 2012; Whitehead et al., 2018; Worley & Barrett, 2001). The heritabilities of floral display characteristics are not well known, and their estimation is complicated by the extent to which they covary with plant size and resource availability. Further research is needed to evaluate the factors that influence floral display and heritabilities.

We found that even within populations, differences in floral display affected the selfing rate for *M. ringens*. This was not limited to populations with large mean display size nor to those with the highest selfing rates (Figure 4). Williams' study of *Delphinium barbeyi* (2007) also documented display‐related differences in the selfing rate within populations. In that study, total floral displays ranged from 2 to 1,400 flowers per plant, and the selfing rate increased strongly with total flower production. Within‐population differences in selfing rate caused by floral display differences may make it difficult to adequately characterize an entire population from a sample. This complicates efforts to document among‐population variation in selfing rate and increases the need to ensure that samples are representative of the population.

### 4.3 Effects of pollinator visitation rate

Selfing rates were higher in *M. ringens* populations with lower rates of pollinator visitation. Decreased visitation is often associated with higher selfing (Kalisz et al., 2004, Yin et al., 2016; but see Koski et al., 2019). There are several possible explanations for this effect that may apply in our system. First, low visitation might increase the opportunity for autonomous selfing in unvisited flowers (Dole, 1990; Goodwillie & Weber, 2018). Second, low pollinator visitation may reduce stigmatic pollen loads, allowing less opportunity for female plants to screen out self‐pollen and selfed offspring (Christopher et al., 2019; Cruzan & Barrett, 2016; Williams & Mazer, 2016). Third, lower pollinator activity often decreases interplant pollinator movements because floral rewards are not depleted, and when rewards are high, pollinators make fewer interplant movements (Dukas & Real, 1993; Heinrich, 1979; Kadmon & Shmida, 1992), so that more geitonogamous self‐pollen is delivered (Karron et al., 2009). One could test these hypotheses by comparing the selfing rates between open‐pollinated and bagged flowers that receive no visits, or compare selfing rates between flowers that receive different numbers of pollinator visits. Identifying the cause of the negative correlation between selfing and visitation will help elucidate the mechanisms responsible for among population selfing rate variation.

We also found substantial differences among populations in the functional composition of floral visitors (Figure 3). Large pollinators like bumblebees appear to be the most effective pollinators, as they visited early while stigmas were open, even though these visitors declined after mid‐morning. Small visitors, while abundant, did not visit early when stigmas were open, and thus appear to be acting as pollen parasites (see Lau & Galloway, 2004; Thomson & Thomson, 1992). Furthermore, like Koski et al., (2017), we found that pollen limitation was more likely at sites where large bee pollinators were less abundant. It is possible that small bees may provide a failsafe pollination mechanism when large bee visitation fails (e.g. populations STR, LIB, LNB). However, the effectiveness of small bees and lepidopterans at transferring pollen, whether self or outcross, has not yet been quantified for *M. ringens*. The variation that we found in the functional groups of floral visitors is most likely due to ecological factors such as habitat type, the relative abundance and composition of coflowering species and landscape context (Cranmer et al., 2012; Herrera, 2020; Mitchell et al., 2009; Primack & Inouye, 1993).

Selfing rate was negatively related to population density in our study, suggesting that geitonogamy decreased when population density was high. Indeed, previous work in experimental populations of *M. ringens* (Karron et al., 1995; Karron et al., 1995) showed that pollinators tended to move more frequently between plants at high density, leading to increased cross‐pollination.

### 4.4 No effects of floral morphology

Although there was substantial among‐population variation in heritable (Christopher et al. in prep.) floral traits in our study (e.g. herkogamy, flower size), these were not associated with the selfing rate. Several other studies have found strong relationships between selfing and herkogamy (Brunet & Eckert, 1998; Herlihy & Eckert, 2005; Brunet & Sweet, 2006; Medrano et al. 2012). Indeed, small anther‐stigma distance is often used as a reliable indicator of selfing, even in the absence of confirmation from progeny testing (Brys et al., 2013; Brys & Jacquemyn, 2012; Gamble et al., 2018; Opedal, 2018). We had expected to find a strong association between selfing and herkogamy in this study, since a prior investigation of variation among *M. ringens* individuals in an experimental garden showed a strong relationship (Karron et al., 1997), and our current study included substantial interpopulation variation in floral morphology. These previous studies examined the herkogamy‐selfing rate association within one population at the individual level, and the extent to which results from comparison of individuals within a population can be scaled up to differences among populations is difficult to assess (Herlihy and Eckert, 2004; Herlihy & Eckert, 2005).

Although herkogamy is sometimes used as a proxy for the mating system, the two are not always correlated. Populations of *Aquilegia canadensis* showed no interpopulation association between herkogamy and the mating system, despite significant differences in floral morphology, including herkogamy (Herlihy & Eckert, 2005). Herlihy and Eckert hypothesized that the effect of herkogamy is obscured at larger spatial scales, so that population‐level factors such as population size and density are more important than an individual‐level trait like herkogamy, which varies substantially within populations. In our study, 57% of the variation in herkogamy is within populations. Similarly, Koski et al., (2018) found that herkogamy was not associated with autonomous selfing in *Campanula americana;* instead, reduced dichogamy was the main factor determining autonomous self fruit production. In *M. ringens*, most of the selfing was probably caused by geitonogamy due to large floral displays. Since herkogamy does not affect geitonogamy, it did not play a key role in interpopulation variation in selfing for our work. Our study and others that do not show a pattern related to herkogamy serve to emphasize the fact that without direct measurement of the selfing rate, one cannot automatically assume that herkogamy or other floral traits are reliable indicators of the expression of the mating system.

## CONFLICT OF INTEREST

None declared.

### PEER REVIEW

The peer review history for this article is available at https://publons.com/publon/10.1111/jeb.13781.

## Supporting information

Table S1‐S3

## Data Availability

Data are available from the Dryad Digital Repository: https://doi.org/10.5061/dryad.v9s4mw6v5.
