# Supplementary material for: Selfing rates vary with floral display, pollinator visitation and plant density in natural populations of Mimulus ringens
Source: J Evol Biol. 2021 Mar 27;34(5):803–15. doi: 10.1111/jeb.13781 (PMC8252063; doi:10.1111/jeb.13781)
Supplement: Supplementary file 1 — Table S1‐S3 [file JEB-34-803-s001.docx]

**Supplemental Table 1.** Locations for the populations used in this study.

| **Population** | **Code** | **County** | **Latitude** | **Longitude** |
| --- | --- | --- | --- | --- |
| Cull Barn  Cuyahoga Valley National Park | CBC | Summit | 41.252771 | -81.548392 |
| Wolf Creek Environmental Center  Medina County Parks | ECM | Medina | 41.116670 | -81.737585 |
| Hinckley Reservation Beaver Pond  Cleveland Metroparks | HBP | Medina | 41.206317 | -81.691658 |
| Lake Erie Bluff Park  Lake County Metroparks | LEB | Lake | 41.785655 | -81.183065 |
| Liberty Park  Summit County Metro Parks | LIB | Summit | 41.29468 | -81.401495 |
| Lucia Nash Beaver Pond  The Nature Conservancy | LNB | Geauga | 41.438804 | -81.180091 |
| Mosquito Creek Wildlife Area  Ohio Division of Natural Resources | MCW | Trumbull | 41.425646 | -80.817777 |
| Morgan Swamp Beaver Pond  The Nature Conservancy | MSB | Ashtabula | 41.643450 | -80.892086 |
| Rising Valley Park  Cleveland Metroparks | RIS | Medina | 41.259202 | -81.694408 |
| SKOK Meadow  Lake County Metroparks | SKO | Lake | 41.657295 | -81.191728 |
| Strongsville Wildlife Area  Cleveland Metroparks | STR | Cuyahoga | 41.318994 | -81.809941 |
| Windhover Bog  Bath Nature Preserve | WBW | Summit | 41.176049 | -81.647164 |
| Wetmore Road  Cuyahoga Valley National Park | WET | Summit | 41.21808 | -81.55386 |

**Supplemental Table S2.** Correlations among *Mimulus ringens* floral traits. All correlations are significant (P<0.01), N=364 flowers.

|  | Corolla Width | Corolla Height | Tube Length | Style Length |
| --- | --- | --- | --- | --- |
| Corolla Height | 0.492 |  |  |  |
| Tube Length | 0.514 | 0.510 |  |  |
| Style Length | 0.338 | 0.350 | 0.721 |  |
| Herkogamy | -0.180 | -0.146 | -0.176 | 0.242 |

**Supplemental Table S3.** Floral display sizes *±* SE for the large and small display groups within populations. N=10 for each entry.

| Population | Floral display group | Mean display |
| --- | --- | --- |
| CBC | high | 46 ± 5 |
|  | low | 17 ± 2 |
| ECM | high | 25 ± 4 |
|  | low | 11 ± 1 |
| HBP | high | 29 ± 4 |
|  | low | 14 ± 1 |
| LEB | high | 70 ± 17 |
|  | low | 20 ± 2 |
| LIB | high | 105 ± 21 |
|  | low | 28 ± 3 |
| LNB | high | 97 ± 37 |
|  | low | 15 ± 2 |
| MCW | high | 108 ± 31 |
|  | low | 19 ± 2 |
| MSB | high | 126 ± 21 |
|  | low | 43 ± 6 |
| RIS | high | 96 ± 21 |
|  | low | 24 ± 2 |
| SKO | high | 73 ± 9 |
|  | low | 18 ± 2 |
| STR | high | 40 ± 6 |
|  | low | 14 ± 1 |
| WBW | high | 80 ± 15 |
|  | low | 25 ± 9 |
| WET | high | 52 ± 3 |
|  | low | 19 ± 4 |
